# Mineral Composition in Delactosed Dairy Products: Quality and Safety Status

**DOI:** 10.3390/foods11020139

**Published:** 2022-01-06

**Authors:** Rosalia Crupi, Vincenzo Lo Turco, Enrico Gugliandolo, Vincenzo Nava, Angela Giorgia Potortì, Salvatore Cuzzocrea, Giuseppa Di Bella, Patrizia Licata

**Affiliations:** 1 Department of Veterinary Science, University of Messina, Polo SS Annunziata, 98168 Messina, Italy; rcrupi@unime.it (R.C.); plicata@unime.it (P.L.); 2 BioMorf Department, University of Messina, Polo SS Annunziata, 98168 Messina, Italy; vloturco@unime.it (V.L.T.); vnava@unime.it (V.N.); gdibella@unime.it (G.D.B.); 3 Chibiofaram Department, University of Messina, Polo Papardo, 98166 Messina, Italy; salvator@unime.it

**Keywords:** food safety, delactosed dairy products, mineral elements, principal component analysis, daily intake

## Abstract

Mineral elements are ingested through the diet (Li, Be, B, Na, Mg, Al, K, Ti, V, Cr, Mn, Fe, Co, Ni, Cu, Zn, As, Se, Sr, Mo, Ag, Cd, Sb, Ba, Tl, Pb, and Bi). Essential minerals have structural, biochemical, nutritional and catalytic functions; therefore, they are fundamental for human health. In this research, thirty commercial delactosed dairy products from different varieties were supplied by various markets in Sicily (Italy), and their mineral contents were determined by using inductively coupled plasma mass spectrometry (ICP-MS) with the following aims: (1) to highlight the differences among various products; (2) to evaluate if it is possibly related to the analyzed samples of their product group; (3) to evaluate the nutritional quality and safety related to intake of these dairy products. Evident differences were found among the samples depending on the type of product. A good separation between mozzarella—on the one hand—and crescenza and primo sale—on the other—was observed. The mozzarella samples were distinguished by the higher Fe, V and Co contents, and the lower amount of Al. Based on shares of the RDA, the analyzed dairy samples are a good source of Ca (up to 58% of the nutrient reference values), with a relatively high concentration of Na (between 5.5% and 22%). Any safety risk for consumers due to exposures to toxic elements through analyzed samples is excluded. The obtained results give reason to expect further insight concerning the direct comparison between the delactosed and non-delactosed product, in order to evaluate if the manufacturing process can affect the content of some mineral.

## 1. Introduction

Around 75% of the population (elderly, young and newborns) worldwide have limited expression of the lactase enzyme. Lactase deficiency can lead to lactose intolerance, and the consumption of milk and dairy products in people with this disorder causes abdominal bloating and very severe stomach pain. Fortunately, there are many lactose-free dairy products on the market today. In vivo, lactase (β-D-galactosidase) is an enzyme secreted by intestinal villi that hydrolyses the disaccharide lactose into glucose and galactose and is essential for the digestion of bovine milk, which contains an average of 4.8% lactose. Deficiency for this gene leads to malabsorption of lactose and subsequent fermentation of lactose by the gut flora. Lactose intolerance develops when afflicted individuals experience abdominal pain, diarrhea, bloating, flatulence, and other gastrointestinal symptoms following lactose consumption [1]. Since lactose is a disaccharide composed of glucose and galactose, it can be hydrolysed into these monosaccharides using either a β-glucosidase or β-galactosidase [2]. Delactosed dairy products can provide essential nutrients present in milk to lactose-intolerant people. There are dairy products on the market that contain very little or no lactose, and these are generally well tolerated by people who are lactose-intolerant. Delactosed dairy has currently a wide and growing health appeal to all consumers and in countries where most people are lactose-tolerant. Basically, for some kinds of cheeses, the lactose content is lowered due to the action of lactic acid bacteria during ripening. Hence, in general, aged, hard cheeses such as Parmesan, Cheddar or Swiss cheeses will have a very low lactose concentration, while fresh or young cheeses contain a higher lactose content, that can cause a reaction among lactose-intolerant people, depending on the amount that is consumed. Nowadays, the delactosed dairy market is the fastest growing segment in the dairy industry [3]. Mineral elements play important roles in living systems, especially in the regulation of enzymatic activities, facilitating the membrane transfer of essential nutrients, and maintenance of nervous and muscular irritability, and others. An alteration of the content of these essential minerals can cause disturbances in the entire physiological system [4]. Delactosed products represent a good source of essential elements in human nutrition [5]. Minerals are important for assessing the safety and quality of milk. In fact, elements determination in milk can be a valid tool for assessing the presence of metal residues that can be indicative of the health status in milk and an indirect indicator of pollution in the environment in which the food produced is found [6]. The content of mineral elements in milk can be linked to the feed, water and drugs administered to the animal. Dairy products are rich in essential nutrients for good bone health, especially for their calcium and potassium content [7]. Proper calcium intake affects skeletal calcium and retention during growth [8]. The high calcium levels play an important role in bone development, strength and density for children and in the prevention of osteoporotic fractures in the elderly [7,8]. Some studies show that frequent consumption of dairy products and milk serves to prevent periodontal disease [9]. In addition, calcium is useful in reducing cholesterol absorption and control of body weight and blood pressure. In order to determine and visualize the differences among the samples and the relationship between observations and variables, a multivariate statistical analysis called principal component analysis (PCA) is often applied to experimental data [10,11,12,13]. In this work, the determination of concentration of mineral elements by ICP-MS in commercial delactosed dairy products from various markets in Sicily is described. The first aim of the research was to evaluate the element content to highlight the differences among various products. Principal Component Analysis (PCA) was applied to mineral element contents in order to correlate the samples to their product group. The second aim was to evaluate the elements contribution derived by ingestion of delactosed dairy samples under analysis and to compare the obtained values with the values of the Recommended Dietary Allowance (RDA) and Adequate Intake (AI) for essential trace elements. In addition, toxic elements exposures were evaluated in order to compare toxic with tolerable daily intake (TDI), or tolerable weekly intake (TWI), or benchmark dose lower confidence limit (BMDL01).

## 2. Materials and Methods

### 2.1. Sample Collection

In this study, a total of 30 commercial delactosed dairy product samples of 10 different varieties (yogurt, cottage cheeses, crescenza, robiola, primo sale, cream cheeses, processed cheeses, processed sliced cheeses, pasta filata, and mozzarella) were supplied by various markets in Sicily, a region of Southern Italy. Samples were randomly selected according to store availability and consumers’ preferences. All selected cheeses were made from cow milk.

### 2.2. Sample Preparation

The methods followed those described in Potortì et al. [13]. About 0.5 g of each sample was accurately weighed into PTFE vessels, added with 1 mL of internal Re standard at 0.5 mg/L bought from Fluka (Milan, Italy), and then was digested with 8 mL of HNO_3_ (65%, *v*/*v*) and 2 mL of H_2_O_2_ (30%, *v*/*v*) purchased from Fluka (Milan, Italy). Instrumental parameters and settings are reported in Table 1.

Allowed to cool, each sample was made up to volume with ultrapure water (25 mL) bought from Merck (Darmstadt, Germany). Blank solution and certified reference materials were digested under the same conditions of the samples. The samples were filtered with 0.45 µm PTFE filters, purchased from Waters (Milford, MA, USA), to delete the larger particles. All determinations were carried out in triplicate.

### 2.3. Instrumentation

Samples were digested in triplicate using a closed-vessel microwave digestion system Ethos 1 (Milestone, Bergamo, Italy) equipped with sensors for temperature and pressure control and provided with PTFE (plytetrafluoroethylene) vessels capable of withstanding pressures of up to 110 bar. The determination of Li, Be, B, Na, Mg, Al, K, Ti, V, Cr, Mn, Fe, Co, Ni, Cu, Zn, As, Se, Sr, Mo, Ag, Cd, Sb, Ba, Tl, Pb, and Bi was carried out by ICP-MS iCAP-Q (Thermo Scientific). The ICP torch was a classic Fassel-type torch with a wide diameter (2.5 mm) fitted with a shield torch system. ICP-MS was equipped with an autosampler ASX520 (Cetac Technologies Inc., Omaha, NE, USA) and an integrated sample introduction system. A standard cyclonic spray chamber made of PFA with a nebulizer with a 6 mm outside was included. The instrument has a system that produces acceleration ions via an initial ion lens stack into the RAPID (Right Angle Positive Ion Deflection) lens that efficiently deflects analyte ions by 90° before entry into the QCell. Ni sampler and skimmer cones of 1.1 mm and 0.5 mm were utilized. A Qcell system of collision/reaction quadrupole with helium gas was used to minimize polyatomic interferences arising from plasma and matrix (KED mode).

### 2.4. Multielement Analysis by ICP-MS 

The instrument is operated in KED mode. Monitored isotopes were: 7Li, 9Be, 11B, 23Na, 24Mg, 27Al, 39K, 48Ti, 51V, 52Cr 55Mn, 56Fe, 59Co, 60Ni, 63Cu, 66Zn, 75As, 80Se, 88Sr, 98Mo, 107Ag, 114Cd, 121Sb, 138Ba, 205Tl, 208Pb and 209Bi. Integration times were 0.01 s/point for Mg, Na and K, 0.5 s/point for As, V, Se and Fe, 0.1 s/point for other elements. Three replicate acquisitions were taken. The operating conditions of ICP-MS analysis are reported in Table 2.

## 3. Results

### 3.1. Method Validation

The method was validated according to Eurachem criteria [14]. Table 3 summarizes the results of the method validation. For the analyzed elements, the evaluation of the linearity was based on injections of the 5 standard solutions. Each solution was injected six times (*n* = 6). Good linearity was observed in each concentration range, with R^2^ values ranged from 0.9972 (for K) to 0.9998 (for Li, V, Mn, Co, Ni, Sr, Sb and Pb). The limits of detection (LODs) ranged from 0.001 to 1323 μg kg^−1^, and limits of quantification (LOQs) ranged from 0.003 to 4366 μg kg^−1^ and were experimentally calculated as 3.3 s/S and 10 s/S, respectively, where s is the standard deviation of the response of ten blanks and S is the slope of the calibration curve. The lowest average recovery was observed for sodium with 85.29%, while the highest was obtained for titanium and cobalt with 101.00%. Accuracy was assessed evaluating six determinations over skimmed milk powder certified reference material (ERM-BD150) and was reported as the percent recovery between the value found with the calibration curve and the true value reported in the certified reference materials. When the element was not certified in the reference material, the matrix was spiked with the known amount of analyte and was analyzed following the procedures discussed before. Based on these results, the analytical characteristic (linearity, sensitivity, accuracy and precision) can be considered satisfactory for the aims of the analysis.

### 3.2. Element Contents in Commercial Delactosed Dairy Products from Different Varieties

In Table 4, the amounts of elements content in commercial delactosed dairy products from the 10 different varieties are listed. The concentrations observed in the various samples analyzed were in line with those reported in the literature in samples of dairy products with lactose [15]. Among essential elements, Na concentration was the highest (with max mean value of 10,473 mg/kg in processed sliced cheeses), followed by Ca (pasta filata cheeses: mg/kg), K (primo sale cheeses: 252 mg/kg) and Mg (pasta filata cheeses: 93.84 mg/kg). Sodium concentrations found in delactosed dairy products were higher than the values found by Zamberlin et al. [5] in yogurt and ice cream samples (80 mg/100 g and 69 mg/100 g) but in line with cheese samples (Brie, Cheddar, Cream Cottage, Edam, Feta, Gouda, Parmigiano, Stilton). The samples studied in this work were also higher than the plain yogurt and yogurt with added fruit found by Khan et al. [16]. The contribution of delactosed dairy products that contain added amounts of salt can provide important sources of sodium. The calcium levels determined in these samples were higher than yogurt and ice cream samples (200 and 130 mg/100 g, respectively) shown by Zamberlin et al. [5], but the concentrations of calcium in the present study were approximately the same as reported for cheese samples (Brie, Cheddar, Cream Cottage, Edam, Feta, Gouda, Parmigiano, Stilton). The concentrations of calcium were higher compared to dairy products showed by Khan et al. [16] in plain yogurt and yogurt with added fruit with values of 1259 and 1304 mg/kg respectively. The delactosed derivatives therefore represent a very rich source of calcium. Furthermore, the absorption of this important element also depends on the level of vitamin D and the age of a person. At the same time, it has been shown that the bioavailability of the calcium contained in the products is better than that of other sources. As for potassium, it is one of the most important intracellular cations. It has been determined that potassium intake has a positive effect on human bones [17]. The potassium concentrations evaluated in this work were higher in the primo sale cheeses than in all the other delactosed samples. These values were also in line with those reported in literature by Zamberlin et al. in the yogurt and ice cream samples with concentrations equal to 280 and 160 mg/100 g, respectively, but lower than cheese samples (Brie, Cheddar, Cream Cottage, Edam, Feta, Gouda, Parmigiano, Stilton). Moreover, potassium levels were lower in all free-lactose dairy samples than reported by Khan et al. [16] in plain yogurt and yogurt with added fruit. By comparison with the published literature, the concentrations of magnesium in the present study were lower than those reported by Zamberlin et al. [5] for yogurt and ice cream (19 and 13 mg/100 g, respectively) and also lower than cheese samples. Magnesium plays an important role in several physiological processes, such as neuromuscular transmission and muscle contraction, metabolism of proteins and nucleic acids, and bone growth. In Western countries, about 20% of the total magnesium is consumed through the regular consumption of milk and dairy products [17]. However, in our study, the magnesium levels found in the delactosed dairy products were lower than those reported in the literature in dairy products with lactose by Zamberlin et al. [5] and Khan et al. [16]. The concentrations of Se found in our samples were very low and it was contained only in the cream cheese, processed cheese and mozzarella cheese samples. In all the other delactosed products, the selenium concentrations observed were below the instrumental detection limits. Among toxic metals, cadmium showed the highest concentrations in primo sale cheeses (0.029 mg/kg), while the lowest concentrations in processed sliced cheeses and pasta filata cheeses (0.010 mg/kg).

Based on Regulation No. 1881/2006, maximum permitted levels (MPL) for toxic metals in dairy products were not fixed, excluding for Pb in milk (0.02 mg/kg) [18]. The Pb in samples examined shows remarkably low levels in most of the samples analyzed (nd-0.009 mg/kg). However, the toxic metal levels observed do not foresee health risks from the consumption of these products. However, for greater food safety, besides continuous surveillance, it would be advisable to establish maximum limits for metals either in milk and in dairy products. Based on the preliminary data obtained in this work, it can be assumed that the consumption of delactosed dairy products can provide a valuable content of essential elements important from a nutritional point of view and for various metabolic activities. Furthermore, as regards the concentrations of toxic elements, the latter are not at toxicological risk for the consumer, because the concentrations found in various samples were below the detection limits.

### 3.3. Principal Component Analysis

**Figure 1:**
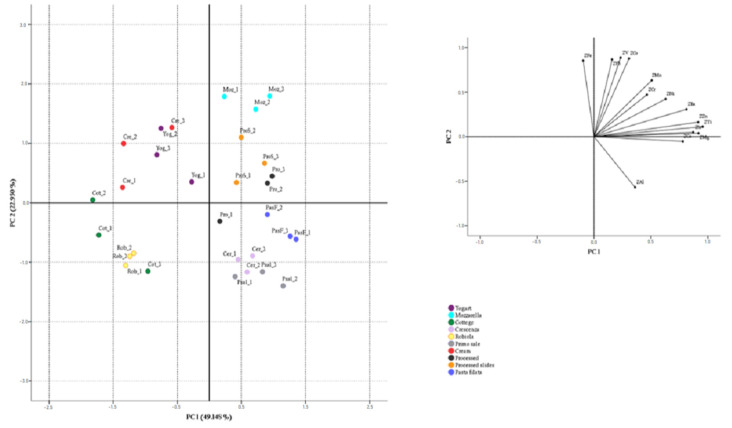
PCA for delactosed dairy samples. Inserts: loadings plot for PC1 and PC2.

Results of element concentrations in samples were subjected to a Factor Analysis by Principal Components extraction. Principal Components Analysis (PCA) was applied to the data matrix having 18 variables (Ca, Na, K, Mg, Fe, Al, Zn, Ti, Sr, Mn, Ba, Ni, Cd, V, Cr, Hg, Co and Pb) and 30 dairy samples. First of all, the suitability of data for PCA was checked. The Kaiser–Meyer–Olkin test revealed a value of 0.628 and the Bartlett’s Test of Sphericity (546.653) reached statistical significance (*p* < 0.001), showing that the correlation matrix is factorable and appropriate for Factor Analysis. Therefore, the data set was subjected to PCA. Four principal components with eigenvalues exceeding one (7.133, 3.865, 1.805 and 1.374) were extracted, according to a Kaiser Criterion. The extracted components explained up to 78.758% of total variance (39.627%, 21.471%, 10.28% and 7.632%, respectively). From the matrix component, the variables with low saturation in the first two factors were identified (they were K, Hg, Na and Cd) and removed from the data set. After that, the PCA was repeated using only the other variables and a Varimax rotation. So, two principal components with eigenvalues exceeding one (5.798 and 4.296) were extracted and the percentages of the total variance in rotated model were 41.412% and 30.686%, respectively. The first component reported the highest positive correlation with Ti (0.955), Mg (0.917) and Zn (0.915); the second component showed the highest positive correlation with V (0.887), Co (0.977), Pb (0.867) and Fe (0.853), while a negative correlation can be observed for Al (−0.568). Figure 1 shows the PC1/PC2 score plot, where a slight tendency to groupings among the samples of the same category can be observed. PC1 seems to split between yogurt, cream, cottage and robiola samples and the rest of the cheeses studied. The first ones showed always negative PC1 scores, while the rest showed positive PC1 scores. The cream and yogurt samples, on the other hand, were separated from the robiola samples on PC2. With respect to samples with positive PC1, a good separation between the mozzarella and the crescenza and primo sale group can be observed. Mozzarella samples had the highest positive scores on PC2, having higher Fe, V and Co levels and a lower amount of Al than the others.

### 3.4. Elements Uptake by Delactosed Dairy Products

Table 5 showed the assessments of elements contribution derived by ingestion of delactosed dairy products under analysis. The daily exposures (mg/d) were estimated by multiplying the mean concentrations (mg/kg) in samples by the quantity consumed (g food/d). The calculations were made using the quantity reported on the package for a single serving. They were: 125 g for yogurt and mozzarella; 80 g for crescenza, robiola and primo sale; 75 g for cottage cheese; 45 g for pasta filata; 40 g for cream cheese; 28.6 g (1 piece) for processed sliced cheese; 20.5 g (1 piece) for processed cheese. The obtained values were compared with the values of the Recommended Dietary Allowance (RDA) and Adequate Intake (AI) for essential trace elements [19,20]. The highest RDA shares (between 6% and 58%) were found for calcium, followed by sodium (between 5.5% and 22%). Iron, chromium, and magnesium uptake reached the maximum values in mozzarella (6.19%, 6.7% and 1.4%, respectively), whereas potassium uptake reached the maximum values in yogurt (1.3%). Selenium and copper were determined for mozzarella and processed cheese samples, and for only one sample of cream cheese; RDA shares were, respectively, 15.8% and 3% for mozzarella, 0.7% and 0.1% for processed cheese, and 2.6% and 0.4% for the samples of cream cheese. Toxic element exposures (calculated for an adult of 60 kg) were compared with tolerable daily intake (TDI), or tolerable weekly intake (TWI), or benchmark dose lower confidence limit (BMDL01) [21,22,23,24,25,26]. The results highlight that the values are not worrisome (maximum value: 3.6% for lead in mozzarella).

## 4. Conclusions

To date, there are very few works in the literature for samples of delactosed dairy products. Evident differences were found among the samples depending on the type of product. The mozzarella samples were distinguished by the higher Fe, V and Co contents, and the lower amount of Al. Further studies have been undertaken by our research group and are in any case necessary for a greater number of delactosed and non-delactosed samples to observe if there are significant differences between various types of dairy products. For greater food safety, it would be appropriate to establish ML for all mineral elements for milk and dairy products. Our data show that the delactosed samples present a very low toxicological risk and, in particular, the delactosed cheeses represent a good alternative to derivatives with lactose, especially for subjects who have a lactose intolerance, but also for the organoleptic characteristics of the products and for some nutritional aspects.

## Figures and Tables

**Table 1:** Operative conditions for the microwave oven digestion.

Step	Time	Temperature	Microwave Power
1	15 min	0–180 °C	1100 W
2	15 min	180 °C	1100 W
3	20 min	Cooling	

**Table 2:** Conditions of ICP-MS.

Spectrometer	iCAP Q Thermo Scientific with Qcell Reaction System
Nebulizer	Concentric PFA
RF generator	1550 W
Sample depth	5 mm
Interface	Sample and skimmer cones in Ni
Interface pressure	1.89 × 10^+00^ Pa
Argon flow (plasma/ausiliario/carrier)	14/0.8/1.1 L/min
Sample introduction Flow	0.93 mL/min
Scanning condition	Number of replicates: 3, dwell time: 1 s
CCT gas flow (He)	4.7 mL/min
Vacuum	<7, 5 × 10^−7^ Pa
Extract Lens 1 Voltage	1.5 V
Spray chamber temperature	2.7 °C

**Table 3:** Analytical characteristics of method.

SKIMMED MILK POWDER ERM-BD150 (mg/kg)						
Element	LOD (μg/kg)	LOQ (μg/kg)	R^2^	Experimental Value	Expected Value	Recovery (%)
Li	0.003	0.010	0.9998	1.90 *	2.00 *	95.00
Be	0.005	0.017	0.9997	1.92 *	2.00 *	96.00
B	0.003	0.010	0.9997	1.89 *	2.00 *	94.50
Na	1.323	4.366	0.9975	3565	4180	85.29
Mg	0.037	0.122	0.9991	1197	1260	95.00
Al	0.081	0.267	0.9995	1.91 *	2.00 *	95.50
K	0.221	0.729	0.9972	15,106	17,000	88.86
Ti	0.001	0.003	0.9997	2.02 *	2.00 *	101.00
V	0.001	0.003	0.9998	2.01 *	2.00 *	100.50
Cr	0.001	0.003	0.9997	1.97 *	2.00 *	98.50
Mn	0.001	0.003	0.9998	0.285	0.289	98.62
Fe	0.014	0.046	0.9997	4.35	4.60	94.57
Co	0.001	0.003	0.9998	2.02 *	2.00 *	101.00
Ni	0.018	0.054	0.9998	1.96 *	2.00 *	98.00
Cu	0.015	0.050	0.9973	1.03	1.08	95.37
Zn	0.057	0.188	0.9982	43.95	44.80	98.10
As	0.001	0.003	0.9997	2.00 *	2.00 *	100.00
Se	0.062	0.205	0.9980	0.179	0.188	95.21
Sr	0.004	0.013	0.9998	1.98 *	2.00 *	99.00
Mo	0.002	0.007	0.9997	2.01 *	2.00 *	100.50
Ag	0.001	0.003	0.9997	1.98 *	2.00 *	99.00
Cd	0.001	0.003	0.9997	0.0108	0.0114	94.74
Sb	0.001	0.003	0.9998	1.92 *	2.00 *	96.00
Ba	0.002	0.007	0.9997	1.95 *	2.00 *	97.50
Tl	0.001	0.003	0.9997	1.92 *	2.00 *	96.00
Pb	0.001	0.003	0.9998	0.018	0.019	94.74
Bi	0.001	0.003	0.9997	1.97 *	2.00 *	98.50
Hg *	0.001	0.003	0.9997	0.061	0.060	101.67

* Not present in the certified matrix. Added later to the matrix.

**Table 4:** Element contents (mg/kg) in 10 different varieties of Italian dairy products (mean ± standard deviation).

	Yogurt (*n* = 3)		Cottage (*n* = 3)		Crescenza (*n* = 3)		Robiola (*n* = 3)		Primo Sale (*n* = 3)		Cream (*n* = 3)		Processed (*n* = 3)		Processed Slices (*n* = 3)		Pasta Filata (*n* = 3)		Mozzarella (*n* = 3)	
	Mean	S.D.	Mean	S.D.	Mean	S.D.	Mean	S.D.	Mean	S.D.	Mean	S.D.	Mean	S.D.	Mean	S.D.	Mean	S.D.	Mean	S.D.
Ca	1223	25.2	2843	121.0	3567	58.6	807	51.6	4250	90.0	1114	132.6	3799	235.3	6011	116.8	6145	126.8	3700	180.3
Na	657	144.8	3217	231.8	3067	61.5	3144	368.8	4117	1732.6	3344	417.6	9461	851.1	10,473	802.6	4722	818.4	2495	447.2
K	213	6.6	95	18.6	170	18.3	197	28.2	252	13.6	193	17.0	221	36.0	98	5.9	194	21.3	65	14.4
Mg	31.42	13.94	11.05	3.87	36.66	6.89	13.17	2.50	46.74	10.37	20.09	10.22	33.72	8.39	45.49	8.80	93.84	10.24	40.74	18.62
Fe	2.58	1.12	0.38	0.28	0.36	0.12	0.52	0.24	0.04	0.02	1.12	0.26	0.24	0.12	2.29	1.04	0.61	0.25	6.94	1.45
Al	0.43	0.24	0.25	0.18	0.57	0.08	0.55	0.13	0.34	0.12	0.16	0.09	0.48	0.22	0.43	0.27	0.86	0.09	0.13	0.01
Zn	0.33	0.10	0.40	0.26	1.31	0.04	0.26	0.15	1.64	0.63	0.46	0.27	2.42	1.36	1.47	0.19	2.07	0.33	3.61	0.92
Ti	0.75	0.17	0.26	0.06	1.61	0.38	0.31	0.06	2.15	0.84	0.47	0.19	1.12	0.13	2.08	0.49	2.33	0.47	1.89	0.52
Sr	0.14	0.06	0.03	0.02	0.63	0.20	0.12	0.02	0.30	0.06	0.13	0.05	0.41	0.18	0.30	0.03	0.46	0.06	0.49	0.31
Mn	0.043	0.015	0.027	0.015	0.047	0.009	0.022	0.013	0.039	0.008	0.037	0.023	0.061	0.035	0.343	0.422	0.049	0.025	0.191	0.069
Ba	0.027	0.012	0.005	0.003	0.040	0.010	0.011	0.004	0.036	0.018	0.014	0.012	0.037	0.003	0.108	0.014	0.083	0.029	0.206	0.130
Ni	0.016	0.003	0.008	0.004	0.009	0.001	0.012	0.002	0.026	0.009	0.014	0.006	0.042	0.011	0.018	0.008	0.023	0.006	0.051	0.025
Cd	0.018	0.008	0.028	0.024	0.018	0.003	0.025	0.005	0.029	0.013	0.016	0.006	0.020	0.003	0.010	0.001	0.010	0.000	0.023	0.003
V	0.009	0.001	0.001	0.001	0.001	0.001	0.001	0.000	0.001	0.001	0.005	0.003	0.004	0.003	0.011	0.001	0.004	0.004	0.016	0.005
Cr	0.010	0.002	0.007	0.003	0.018	0.002	n.d.	-	0.003	0.001	0.014	0.008	0.043	0.030	0.012	0.008	0.017	0.006	0.021	0.009
Co	0.002	0.001	n.d.	-	n.d.	-	n.d.	-	n.d.	-	0.001	0.001	0.001	0.001	0.003	0.001	0.001	0.000	0.004	0.001
Pb	0.002	0.001	n.d.	-	n.d.	-	n.d.	-	n.d.	-	0.005	0.002	0.004	0.003	0.003	0.001	n.d.	-	0.009	0.005
As	n.d.	-	n.d.	-	n.d.	-	n.d.	-	n.d.	-	0.001	0.000	0.001	0.001	0.002	0.001	n.d.	-	0.002	0.001
Hg	n.d.	-	0.010	0.002	0.012	0.003	0.005	0.001	0.003	0.001	0.007	0.003	0.016	0.004	0.006	0.004	0.031	0.009	0.010	0.002
Se	n.d.	-	n.d.	-	n.d.	-	n.d.	-	n.d.	-	0.036	*	0.018	0.008	n.d	-	n.d.	-	0.069	0.027
Cu	n.d.	-	n.d.	-	n.d.	-	n.d.	-	n.d.	-	0.109	*	0.051	0.019	n.d.	-	n.d.	-	0.241	0.086

* Only one sample positive. n.d., not determined < LOQ.

**Table 5:** Percentage of RDA or IA (A), and of TDI or TWI, or BMDL01 (B) for delactosed cheeses consumption.

	Yogurt		Cottage		Crescenza		Robiola		Primo Sale		Cream		Processed		Processed		Pasta Filata		Mozzarella	
	A	B	A	B	A	B	A	B	A	B	A	B	A	B	A	B	A	B	A	B
Ca	19%		27%		36%		8%		43%		6%		10%		21%		35%		58%	
Na	5.5%		16.1%		16.4%		16.8%		22.0%		8.9%		12.9%		20.0%		14.2%		20.8%	
K	1.3%		0.4%		0.7%		0.8%		1.0%		0.4%		0.2%		0.1%		0.4%		0.4%	
Mg	1.0%		0.2%		0.8%		0.3%		1.0%		0.2%		0.2%		0.3%		1.1%		1.4%	
Fe	2.31%		0.20%		0.20%		0.30%		0.02%		0.32%		0.03%		0.47%		0.20%		6.19%	
Zn	0.4%		0.3%		1.0%		0.2%		1.3%		0.2%		0.5%		0.4%		0.9%		4.5%	
Mn	0.3%		0.1%		0.2%		0.1%		0.2%		0.1%		0.1%		0.5%		0.1%		1.2%	
Cr	3.2%		1.3%		3.6%		n.a.		0.5%		1.4%		2.2%		0.9%		1.9%		6.7%	
Se	n.a.				n.a.		n.a.		n.a.		2.6%		0.7%		n.a.		n.a.		15.8%	
Cu	n.a.				n.a.		n.a.		n.a.		0.4%		0.1%		n.a.		n.a.		3.0%	
Al		88.61%																		
Ni		0.15%		0.04%		0.05%		0.07%		0.16%		0.04%		0.07%		0.04%		0.08%		0.48%
Cd		1.5%		1.4%		0.9%		1.3%		1.5%		0.4%		0.3%		0.2%		0.3%		1.9%
Pb		0.7%		n.a.		n.a.		n.a.		n.a.		0.6%		0.3%		0.3%		n.a.		3.6%
As		n.a.		n.a.		n.a.		n.a.		n.a.		0.2%		0.2%		0.3%		n.a.		1.2%
Hg		n.a.		0.3%		0.4%		0.2%		0.1%		0.1%		0.1%		0.1%		0.6%		0.5%

n.a., not available. RDA value (mg/day): 10 for Zn, 14 for Fe, 1 for Cu, 0.040 for Cr, 0.055 for Se, 2 for Mn, 800 for Ca, 2000 for K, 375 for Mg, and 1500 for Na [19,20]; TDI or BMDL_01_ value (µg/kg_b.w._/day): 22 for Ni [26], 0.3 for As [22], 4 for Hg [25], and 0.5 for Pb [23]; TWI value: 2.5 µg/kg_b.w._/week for Cd [24] and 1 mg/kg_b.w._/week for Al [21].

## Data Availability

All data obtained from this study are included in the manuscript.
